# Ticagrelor-Induced Syncope/Bradyarrhythmia

**DOI:** 10.7759/cureus.12874

**Published:** 2021-01-23

**Authors:** VeeraPavan Kotaru, Jagadeesh K Kalavakunta

**Affiliations:** 1 Cardiology, Ascension Borgess Hospital, Kalamazoo, USA

**Keywords:** ticagrelor, bradyarrhythmia, syncope

## Abstract

Ticagrelor (BRILINTA®) is a very commonly used oral antiplatelet agent in acute coronary syndrome and after percutaneous coronary intervention (PCI). It is a reversible, direct inhibitor of the adenosine diphosphate (ADP) P2Y12 receptor. Most of the patients tolerate the drug well but it is known to cause brady arrhythmias and ventricular pauses, the exact mechanism of which is unclear. We present a case of acute coronary syndrome/unstable angina in a 58-year-old Caucasian gentleman requiring cardiac catheterization and PCI with drug eluting stent deployment and syncope following Ticagrelor loading from long ventricular pauses.

## Introduction

Ticagrelor (BRILINTA®) is a widely used oral antiplatelet agent in acute coronary syndrome and after percutaneous coronary intervention (PCI). It is a rapid onset, potent, reversible, direct inhibitor of the adenosine diphosphate P2Y12 receptor. It is known to cause bradyarrhythmias and ventricular pauses. The exact mechanism of Ticagrelor-induced bradyarrhythmia is unclear, although inhibition of adenosine reuptake is proposed as likely due to structural similarities between Ticagrelor and adenosine. In the acute coronary syndrome treated with Ticagrelor, extracellular adenosine concentration is increased by ischemic myocardial release of adenosine and reduced cellular re-uptake. This will cause amplified agonism of the adenosine A1 receptor leading to negative chronotropy at sinoatrial node and negative dromotropy at atrioventricular node. This case report highlights Ticagrelor’s brady-arrhythmic potential even in the absence of baseline conduction disease or concurrent confounding medications.

## Case presentation

**Figure 1:**
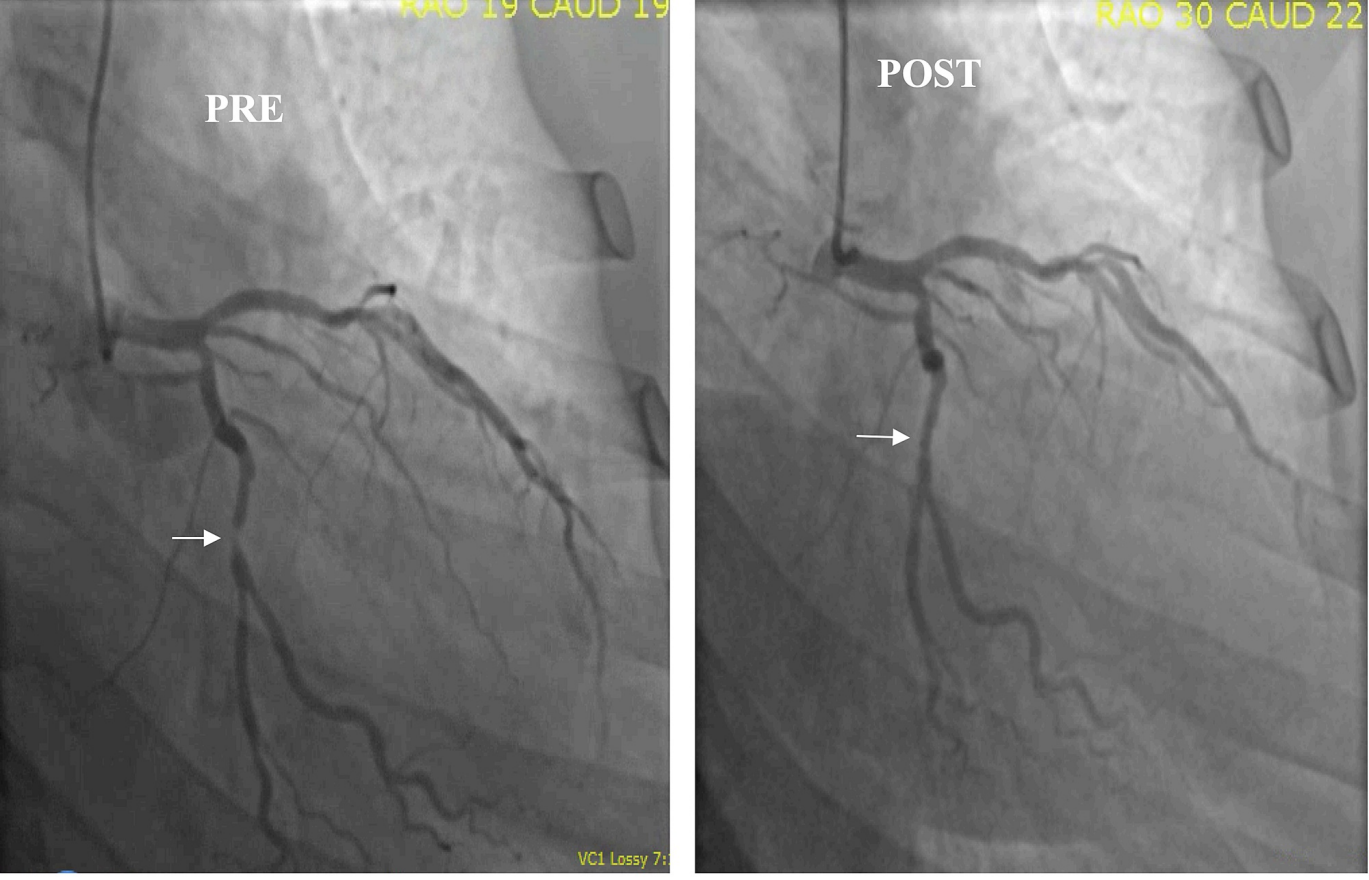
Pre- and post-angiograms of the left circumflex artery in the RAO/CAUDAL view RAO: Right Anterior Oblique

We report a case of a 58-year-old Caucasian man with BMI (Body Mass Index) of 25 with no prior cardiac history or medications presented to our hospital with complaints of chest pain. His pain has been going on for the last few weeks, substernal, pressure like sensation, progressively getting worse with minimal exertion and reached the point where it was limiting his lifestyle. He denies any radiation of the pain and also there was no associated nausea, vomiting or sweating. His father had premature coronary artery disease (CAD). He is not a smoker. Physical exam was unremarkable with stable vitals. Electrocardiogram (ECG) normal sinus rhythm with no acute ischemic changes. Laboratory data revealed troponin elevation at 0.5 ng/dl. Rest of the laboratory data including thyroid stimulating hormone (TSH) was within normal limits. He was diagnosed with non-ST-segment elevation myocardial infarction (NSTEMI) and had cardiac catheterization via radial approach. He was noted to have severe obstructive CAD in the left circumflex artery and underwent stent supported angioplasty with 2.75 x 15 mm XIENCE Sierra™ (Abbott, USA) drug eluting stent successfully without any complications (Figure 1). Right coronary artery was normal angiographically.

**Figure 2:**
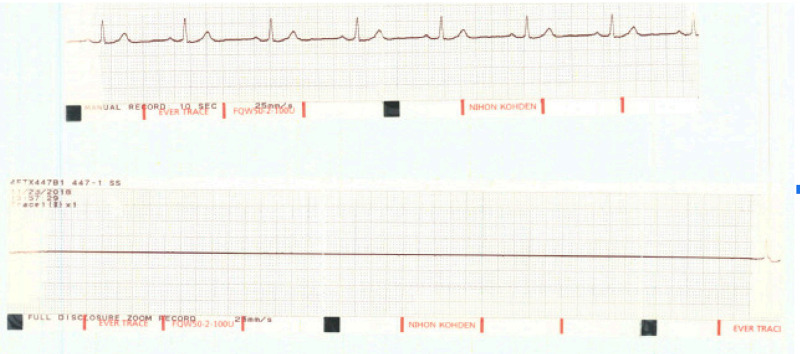
Telemetry strip showing the prolonged pause

At the end of the procedure he was given a loading dose of Ticagrelor 180 mg. He was transported back to the floor in a stable condition. He was at his baseline in the immediate postprocedural period. He was noted to have 3-second ventricular pauses about 90 minutes following the loading dose and in about 2 hours from the loading dose he had an 11-second long pause/asystole causing a syncopal event (Figure 2).

**Figure 3:**
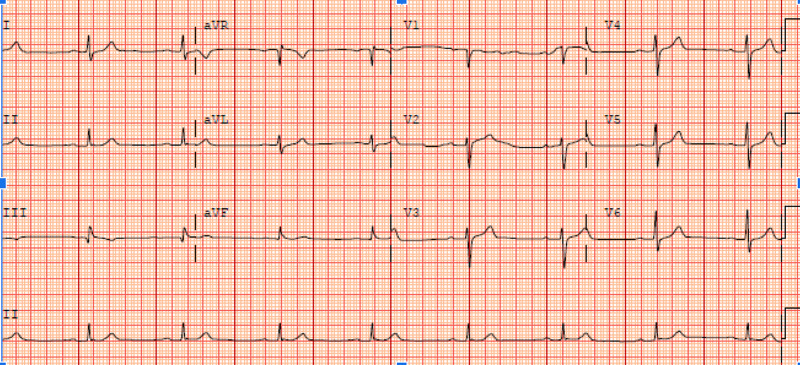
Electrocardiogram showing sinus bradycardia with heart rate of 50 beats per minute and PR interval of 234 msec, First degree AV block

He spontaneously recovered by gaining pulse with normal sinus rhythm. His blood pressure was normal. ECG showed sinus bradycardia with First degree atrioventricular (AV) block but did not show any ST elevation or other abnormalities (Figure 3).

As he woke up he appeared to be confused. His wife was at bedside and she was not quite sure but she thought that he was complaining of some chest discomfort prior to his syncopal event. At this point it was decided to take him back to the catheterization laboratory to make sure he does not have any periprocedural complications including stent thrombosis, though thought to be less likely given no ST elevation on the ECG. Repeat cardiac catheterization showed a widely patent stent. His confusion resolved quickly and had a stroke evaluation which did not reveal any abnormality. At this point it was thought that Ticagrelor was the culprit agent in causing syncope from ventricular pauses. He was started on Prasugrel (Effient®) for antiplatelet needs while continuing aspirin. Echocardiogram revealed normal left ventricular heart function without any valvular abnormalities. He has no history of syncope, snoring or sleep apnea history. He was monitored for 48 hrs which was uneventful and discharged home in stable condition. He also had an outpatient, four weeks of outpatient telemetry monitoring, and tilt table test which did not reveal any abnormalities. Subsequent follow-ups at six and 12 months did not reveal any cardiac issues.

## Discussion

Ticagrelor is an oral, reversible, direct ADP, P2Y12 antagonist with a rapid platelet inhibition capability. It has 36% bioavailability with rapid absorption and reaches peak plasma concentration at 1.5 hrs with loading 180 mg dose [1]. As noted in the PLATO (Platelet Inhibition and Patient Outcomes) trial treatment with Ticagrelor when compared to Clopidogrel in patients with acute coronary syndrome significantly reduced the rate of death from myocardial infarction, vascular causes and stroke. There is no increase in the rate of major bleeding when compared to Clopidogrel [2].

Most of the patients do well with Ticagrelor therapy. Major bleeding, dyspnea, gastrointestinal disturbances and bradyarrhythmias were reported as potential side effects. A subset of the PLATO trial noted that Ticagrelor caused ventricular pauses more than 3 seconds when compared to clopidogrel (6% vs 3.5%) in acute phase of the therapy, but they were infrequent at 30 days (2.2% vs 1.6%) and were rarely associated with any significant symptoms. The pauses were mainly sino-atrial pauses. However, there were no reports of adverse outcomes, no high-grade AV blocks or increased requirement for pacemaker implantation [3]. The exact mechanism of bradyarrhythmias is unclear. Ticagrelor inhibits the uptake of adenosine at the cellular level and potentially increases its plasma concentration which indeed can cause AV blocking effect. There is no clear evidence that it hampers AV conduction.

Ticagrelor is the most likely culprit in our case for causing the ventricular pauses. He did not have any existing conduction abnormalities on the ECG, not on any medications that can cause the bradyarrhythmias, timing of the event correlates with peak plasma concentration of the Ticagrelor and there was no recurrence of pauses on the outpatient follow-ups.

## Conclusions

As noted in our case report significant ventricular pauses and sinoatrial pauses resulting in syncope can occur with Ticagrelor therapy. Though significant adverse outcomes from arrhythmias are not reported in the literature from Ticagrelor therapy, clinicians should promptly recognize and associate this with Ticagrelor therapy in the absence of other causes and switch to an alternative antiplatelet therapy if clinically warranted.
